# Epidemiology of Hospitalized Patients with Babesiosis, United States, 2010–2016

**DOI:** 10.3201/eid2802.210213

**Published:** 2022-02

**Authors:** Evan M. Bloch, Jonathan R. Day, Peter J. Krause, Anne Kjemtrup, Sheila F. O’Brien, Aaron A.R. Tobian, Ruchika Goel

**Affiliations:** Johns Hopkins School of Medicine, Baltimore, Maryland, USA (E.M. Bloch, A.A.R. Tobian, R. Goel);; University of Iowa Health Care, Iowa City, Iowa, USA (J.R. Day);; Simmons Cancer Institute at Southern Illinois University, Springfield, Illinois, USA (J.R. Day, R. Goel);; Yale School of Public Health and Yale School of Medicine, New Haven, Connecticut USA (P.J. Krause);; California Department of Public Health, Sacramento, California, USA (A. Kjemtrup);; Canadian Blood Services, Ottawa, Ontario, Canada (S.F. O’Brien)

**Keywords:** babesiosis, Babesia, epidemiology, United States, hospitalization, parasites, vector-borne infections, ticks

## Abstract

*Babesia* spp. are tickborne parasites that cause the clinical infection babesiosis, which has an increasing incidence in the United States. We performed an analysis of hospitalizations in the United States during 2010–2016 in which babesiosis was listed as a diagnosis. We used the National Inpatient Sample database to characterize the epidemiology of *Babesia*–associated admissions, reflecting severe *Babesia*-related disease. Over a 7-year period, a total of 7,818 hospitalizations listed babesiosis as a primary or secondary admitting diagnosis. Hospitalizations were seasonal (71.2% occurred during June–August) and situated overwhelmingly in the Northeast and Midwest. The patients were predominantly male and of advanced age, which is consistent with the expected epidemiology. Despite a higher severity of illness in more than (58.5%), the mortality rate was low (1.6%). Comparison with state reporting data suggests that the number of hospitalized persons with babesiosis increased modestly during the observation period.

## Abstract 2

Analysis of a national database suggests that incidence of hospitalized babesiosis patients stable and deaths are low.

*Babesia* spp. are tickborne intraerythrocytic apicomplexan parasites responsible for the clinical infection babesiosis. *Babesia microti*, the leading cause of human babesiosis, is endemic in the northeastern and north-midwestern United States (*1*). Although infection in immunocompetent adults may be mild or even subclinical, manifesting as a self-limiting viral-like illness (i.e., fever, headache, myalgia, fatigue), risk for severe disease and complications exists in certain patient populations (i.e., the very young, the elderly, persons with asplenia, and others with immunosuppression). Like *Plasmodium* parasites that cause malaria, *Babesia* spp. infect erythrocytes and induce hemolysis. Clinical complications include severe anemia, renal failure, cardiorespiratory failure, and death (*1*). *Babesia* spp. also are readily transmissible by transfusion of infected erythrocytes. Given that anemia is the major indication for erythrocyte transfusion, coupled with the high proportion of patients at high risk for severe disease in the transfused population, transfusion-transmitted babesiosis has a death rate of ≈20% (*1*,*2*).

Reported cases of babesiosis and other tickborne diseases are increasing (*3*–*5*). Postulated reasons for the increase include expansion of the geographic range of tick vector population, increase in deer (and consequent tick) populations, encroachment of humans into *Babesia* zoonotic habitats, climate change, and other ecologic changes that contribute to a rise in incidence of *Babesia* infection (*6*,*7*). Babesiosis was designated a nationally notifiable disease in the United States in 2011, meaning that states where it was reportable were charged to voluntarily notify the Centers for Disease Control and Prevention (CDC) of cases. As of 2015, babesiosis was reportable in 33 states (*8*,*9*). Although an increase in babesiosis cases has been reported, whether the increase includes primarily outpatients, hospitalized case-patients, or both is uncertain. To test whether hospitalized babesiosis patients are increasing, we analyzed hospitalizations in the United States in which babesiosis was listed as a diagnosis, using the National (Nationwide) Inpatient Sample (NIS) database, which offers a representative sampling of US-based hospitals. This analysis enabled characterization of the epidemiology of admissions, reflecting severe *Babesia*-related disease.

## Methods

This study uses 7 years of data (i.e., 2010–2016) from the NIS, the largest publicly available inpatient healthcare database in the United States. The NIS was developed as a federal–state–industry partnership by the Agency for Healthcare Research and Quality for the Healthcare Cost and Utilization Project (HCUP). Data before 2012 used a 20% stratified probability sample of hospitals rather than discharges (*10*). After a redesign in 2012, the NIS adopted a sampling design that uses a stratified probability sample of 20% of all HCUP participating hospital discharges for each calendar year. This sampling scheme is estimated to cover 90%–97% of the US population across the different years (*11*). The unit of analysis is a single hospitalization and not a specific patient; therefore, a single patient may be represented in multiple observations. Observations are self-weighted and calculated by strata, which are defined by census division (categorized as census region before 2012), bed size, location, teaching status, and hospital ownership.

The NIS provides de-identified discharge data without individual patient or hospital-level identifiers. These data include 1 primary or principal diagnosis code, up to 29 secondary diagnosis codes, and up to 15 procedure codes. The principal diagnosis is the primary reason for admission and is coded in the first diagnosis field. The number of diagnoses and associated data elements was increased from 15 to 25 in 2009 and from 25 to 30 beginning in 2014. We have captured this change in our analysis.

Demographic details extracted from the database were age, sex, and race. Hospital-level characteristics were location (urban vs rural), academic designation (teaching vs. nonteaching), and bed size. Hospitals were categorized as small, medium, or large according to the criteria defined by HCUP, which were based on region, urban–rural designation, and teaching status (*12*). Other variables included admission and discharge status, total charges, expected payment source, length of hospital stay, and hospital characteristics. The NIS database uses All Patient Refined Diagnosis Related Groups (APR-DRGs), a validated inpatient classification system that is widely used in the United States to assess severity of illness and risk for death during hospitalization using multiple variables. The risk for death and severity of illness are ranked on a scale of 1 to 4, corresponding to mild, moderate, major, and extreme.

We used diagnosis and procedure codes from the International Classification of Diseases, Ninth Revision, Clinical Modification (ICD-9-CM), from 2010 through the third quarter of 2015. After the third quarter of 2015, billing codes switched from ICD-9-CM to the International Classification of Diseases, Tenth Revision, Clinical Modification (ICD-10-CM). Babesiosis cases were identified by ICD-9 code 088.82 or ICD-10 code B60.0. Transfusion-transmitted babesiosis infections could not be identified independently because there are no specific ICD-9 or ICD-10 codes for that diagnosis. We described demographic and clinical characteristics as counts, percentages, mean (SD) and median (interquartile range) as appropriate. We stratified results into ICD-9 (2010–2015q3) and ICD-10 (2015q4–2016) data. We analyzed the geographic distribution, demographics. and seasonality of *Babesia*-related hospitalizations and stratified and analyzed hospitalizations by the leading regional divisions. We calculated transfusions and incidence of erythrocyte exchanges during admissions as the binomial proportion of encounters during which >1 blood product was issued or erythrocyte exchange was performed. We performed logistic regression to compare the incidence of various clinical co-morbidities and outcomes. All p values were 2-tailed and statistical significance was set at p<0.05. We analyzed data using Stata version 15 (StataCorp LLC, https://www.stata.com), using survey analysis commands applying the sampling weights as determined by HCUP.

We also performed a graphical comparison of the number and incidence of babesiosis cases reported to CDC during 2011–2016 to compare the overall trends in reporting. The CDC data that were included were reported by individual state health departments; cases were reported by the state of residence, which might not have been the state of exposure.

To test specificity, we performed a sensitivity analysis that restricted the assessment to hospitalizations in which babesiosis was listed in the top 5 diagnoses. The analysis also excluded admissions associated with a primary diagnosis of Lyme disease.

Given that the NIS is a de-identified, publicly available dataset, this study was deemed exempt from review from the Johns Hopkins Institutional Review Board. This analysis was conducted in accordance with the HCUP data use agreement guidelines.

## Results

During a 7-year period, babesiosis was listed as an admitting diagnosis for 7,818 hospitalizations, of which 4,648 (59.5%) listed babesiosis as a primary diagnosis and 3,170 (40.5%) as a secondary diagnosis (Table 1). Annual hospitalizations varied by year, from 676 in 2010 to 1415 in 2013 (Figure). For all hospitalizations, the median age of patients was 67 years (interquartile range 55–77 years); 5,001 (64%) of the associated patients were male, and 6,024 (80.1%) were White.

**Table 1:** Characteristics of hospitalized patients for whom babesiosis was listed as an admitting diagnoses, United States, 2010–2016*

Characteristic	All data	ICD-9 data, 2010–2015q3†	ICD-10 data, 2015q4–2016†
Babesiosis as 1 of all diagnoses	7,818 (100)	6,368 (100)	1,450 (100)
Primary diagnosis babesiosis	4,648 (59.5)	3,838 (60.2)	810 (55.9)
Demographic data			
Age, y			
0–1	30 (0.4)	30 (0.5)	NA
18–44	831 (10.6)	641 (10.1)	190 (13.1)
45–64	2583 (33.0)	2178 (34.2)	405 (27.9)
>65	4374 (55.9)	3519 (55.3)	855 (59.0)
Mean (SD)	64.7 (16.0)	64.5 (16)	65.7 (16.4)
Median (IQR)	67 (55–77)	66 (55–76)	68.5 (55–78)
Sex			
M	5,001 (64.0)	4,081 (64.1)	920 (63.4)
F	2,817 (36.0)	2,287 (35.9)	530 (36.6)
Race/ethnicity			
White	6,024 (80.1)	4,899 (80.2)	1,125 (79.8)
African American	245 (3.3)	180 (3.0)	65 (4.6)
Hispanic	503 (6.7)	403 (6.6)	100 (7.1)
Asian/Pacific Islander	240 (3.2)	170 (2.8)	70 (5.0)
Other	509 (6.7)	459 (7.5)	50 (3.5)
Hospital and temporal			
Admission month§		d	
January	25 (0.8)	15 (0.8)	10 (0.7)
February	60 (1.8)	25 (1.4)	35 (2.4)
March	35 (1.1)	25 (1.4)	10 (0.7)
April	51 (1.6)	41 (2.3)	10 (0.7)
May	109 (3.3)	64 (3.5)	45 (3.1)
June	371 (11.4)	171 (9.4)	200 (13.9)
July	1,192 (36.5)	787 (43.1)	405 (28.1)
August	762 (23.3)	437 (24.0)	325 (22.6)
September	269 (8.2)	179 (9.8)	90 (6.3)
October	80 (2.5)	20 (1.1)	60 (4.2)
November	181 (5.5)	46 (2.5)	135 (9.4)
December	130 (4.0)	15 (0.8)	115 (8)
Elective vs. nonelective admissions			
Nonelective	7,452 (95.4)	6,067 (95.3)	1,385 (95.8)
Elective	356 (4.6)	296 (4.7)	60 (4.2)
Region of hospital¶			
Northeast	6,140 (86.0)	4,915 (86.4)	1,225 (84.5)
Midwest	476 (6.7)	371 (6.5)	105 (7.2)
South	375 (5.3)	295 (5.2)	80 (5.5)
West	150 (2.1)	110 (1.9)	40 (2.8)
Division subset of hospitals#			
New England: ME, NH, VT, MA, RI, CT	1,150 (44.1)	480 (41.4)	670 (46.2)
Mid-Atlantic: NY, PA, NJ	1,115 (42.7)	560 (48.3)	555 (38.3)
East North Central: WI, MI, IL, IN, OH	100 (3.8)	40 (3.4)	60 (4.1)
West North Central: MO, ND, SE, NE, KS, MN, IA	65 (2.5)	20 (1.7)	45 (3.1)
South Atlantic: DE, MD, DC, VA, WV, NC, SC, GA, FL	90 (3.4)	35 (3.0)	55 (3.8)
East South Central: KY, TN, MS, AL	10 (0.4)	**	10 (0.7)
West South Central: OK, TX, AR, LA	20 (0.8)	**	15 (1.0)
Mountain: ID, MT, WY, NV, UT, CO, AZ, NM	**	**	¶
Pacific: AK, WA, OR, CA, HI	55 (2.1)	20 (1.7)	35 (2.4)
Hospital bed size**			
Small	2,159 (30.2)	1,659 (29.1)	500 (34.5)
Medium	2,084 (29.2)	1,609 (28.3)	475 (32.8)
Large	2,898 (40.6)	2,423 (42.6)	475 (32.8)
Hospital teaching status**			
Rural	612 (8.6)	527 (9.3)	85 (5.9)
Urban nonteaching	2,488 (34.8)	2,093 (36.8)	395 (27.2)
Urban teaching	4,041 (56.6)	3,071 (54.0)	970 (66.9)

*Values are no. (%) except as indicated. Data are from the NIS, which offers a representative sampling of US-based hospitals. Weighted national estimates are based on data that were collected by individual states and provided to AHRQ. Total number of weighted discharges in the US based on HCUP NIS: 37,352,013 (2010); 36,962,415 (2011); 36,484,846 (2012); 35,597,792 (2013); 35,358,818 (2014); 35,769,942 (2015); 35,675,421 (2016). In 2012, the NIS was redesigned to optimize national estimates. The nationwide statistics in HCUPnet for the years before 2012 were regenerated using new trend weights to permit longitudinal analysis. The regenerated data were posted to HCUPnet on July 2, 2014. HCUP notes that the statistics for the years before 2012 that are currently on HCUPnet will differ slightly from statistics obtained before that date. Information about the NIS redesign and trend weights is available at https://hcupnet.ahrq.gov. For more information about HCUP data. see http://www.hcup-us.ahrq.gov. AHRQ, Agency for Healthcare Research and Quality; HCUP, Healthcare Cost and Utilization Project; ICD-9, International Classification of Diseases, Ninth Revision; ICD-10, International Classification of Diseases, Tenth Revision; NA, not available; NIS, National Inpatient Sample. †Because of the transition from ICD-9 to ICD-10 in October 2015, the data represent 2 time periods. ICD-9 data reflect 2010 through the third quarter of 2015 (2015q3) and ICD-10 data represent the fourth quarter of 2015 (2015q4) through 2016. §Data for 2011–2014 not available. ¶2010 data not available. #Data available only for 2015 and 2016. **Statistics based on estimates with a relative SE (SE/weighted estimate) >0.30 or a total cell count <10 in the NIS are not reliable. These statistics are suppressed per HCUP policies.

**Figure:**
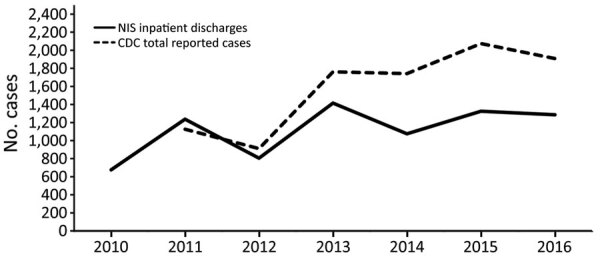
Cases of babesiosis in the United States, 2010–2016, CDC versus NIS data. CDC, Centers for Disease Control and Prevention; NIS, National Inpatient Sample.

Of all hospitalizations, 2,325 (71.2%) occurred in summer (June–August); 6,616 (92.7%) occurred in the Northeast and Midwest. New England (1,150 [44.1%] hospitalizations) and Mid-Atlantic (1,115 [42.7%] hospitalizations) were the leading regions. The admitting hospitals were predominantly urban (6,529 [91.4%]), and admissions were overwhelmingly nonelective (7,452 [95.4%]).

A greater severity of illness was reported in 4,574 (58.5%) hospitalizations; risk for death was assessed as major or extreme for 1,339 (17.2%) hospitalizations (Table 2). The leading complications included acute renal failure (1,594 [20.4%] hospitalizations), respiratory failure (528 [6.8%]), and cardiac failure (270 [3.5%]) (Table 3). A total of 128 deaths occurred over the 7-year period, representing 1.6% of all babesiosis-associated admissions. Babesiosis was the primary hospital-associated diagnosis for 20 of those deaths and secondary for the other 108 deaths. Similar to the distribution of all hospitalizations, most deaths occurred in the Northeast (89 [69.5%]) and the Midwest (10 [7.8%]). 

**Table 2:** Disease severity, risk for death, and concurrent conditions in hospitalizations in which babesiosis was listed as an admitting diagnoses, United States, 2010–2016*

Disease severity and conditions	All data, no. (%)	ICD 9 data, 2010–2015q3,† no. (%)	ICD10 data, 2015q4–2016,† no. (%)
APD-RG severity of illness			
Minor	376 (4.8)	316 (5.0)	60 (4.1)
Moderate	2,863 (36.6)	2,318 (36.4)	545 (37.6)
Major	3,660 (46.8)	2,990 (47.0)	670 (46.2)
Extreme	914 (11.7)	744 (11.7)	170 (11.7)
APD-RG risk for death			
Minor	2,004 (25.6)	1,639 (25.7)	365 (25.2)
Moderate	2,852 (36.5)	2,377 (37.3)	475 (32.8)
Major	2,178 (27.9)	1,718 (27.0)	460 (31.7)
Extreme	779 (10.0)	634 (10.0)	145 (10.0)
Concurrent conditions			
Decreased splenic function or asplenia	560 (7.2)	475 (7.1)	85 (5.9)
HIV‡	20 (0.3)	15 (0.2)	‡
Sickle cell disease	30 (0.4)	30 (0.5)	‡
Lyme disease (any diagnosis)	1,953 (25.0)	1,573 (24.7)	380 (26.2)
Lyme disease (primary diagnosis)	276 (3.5)	221 (3.5)	55 (3.8)
Anaplasmosis and ehrlichiosis	658 (8.4)	548 (8.6)	110 (7.6)
Malaria	52 (0.7)	32 (0.5)	20 (1.4)
Rocky Mountain spotted fever/rickettsial illness	25 (0.1)	20 (0.3)	§
Powassan virus disease, other tick-borne viral encephalitis	§	§	§
Relapsing fever	§	§	§

*Data are from the NIS, which offers a representative sampling of US-based hospitals. APR-DRG, All Patient Refined Diagnosis Related Group; HCUP, Healthcare Cost and Utilization Project; ICD-9, International Classification of Diseases, Ninth Revision; ICD-10, International Classification of Diseases, Tenth Revision; NIS, National Inpatient Sample. †Because of the transition from ICD-9-CM to ICD-10-CM in October 2015, the data represent 2 time periods. ICD-9 data reflect 2010 through the third quarter of 2015 (2015q3), and ICD-10 data represent the fourth quarter of 2015 (2015q4) through 2016. ‡Data from 2011–2014 not available. §Statistics that are based on estimates with a relative SE (SE/weighted estimate) >0.30 or a total cell count <10 in the NIS are not reliable. These statistics are suppressed per HCUP policies.

**Table 3:** Clinical outcomes and healthcare use in patients with babesiosis-associated hospitalizations, United States, 2010–2016*

Clinical outcome	All data	ICD-9 data, 2010–2015q3†	ICD-10 data, 2015q4–2016†
Mortality, no. (%)	128 (1.6)	108 (1.7)	20 (1.4)
Length of stay, d			
Mean (SD)	5.8 (7.3)	5.8 (10.3)	5.8 (6.5)
Median (IQR)	4 (3–7)	4 (2–6)	4 (3–7)
Total hospital charges for primary diagnosis of babesiosis‡			
Mean	$36,850.51	$37,236.39	$36,464.62
Aggregate national bill, USD	$171,281,170	$142,911,768	$29,536,342
Mean national bill per year, USD	$24,468,739	$24,854,221	$23,629,074
Transfusion and apheresis use, no. (%)			
Erythrocyte transfusion	1560 (20.0)	1375 (21.6)	185 (12.8)
Platelet transfusion	208 (2.7)	183 (2.9)	25 (1.7)
Plasma transfused	88 (1.1)	78 (1.2)	10 (0.7)
Erythrocyte exchange	80 (1.0)	75 (1.2)	§
Erythrocyte or plasma exchange	90 (1.2)	75 (1.2)	15 (1.0)
Complications, no. (%)			
Acute renal failure	1,594 (20.4)	1,209 (19)	385 (26.6)
Respiratory failure	528 (6.8)	363 (5.7)	165 (11.4)
Acute heart failure	270 (3.5)	200 (3.1)	70 (4.8)
Disseminated intravascular coagulation	149 (1.9)	129 (2.0)	20 (1.4)

*Data are from the NIS, which offers a representative sampling of US-based hospitals. Weighted national estimates are based on data that were collected by individual states and provided to AHRQ. Total number of weighted discharges in the United States based on HCUP NIS: 37,352,013 (2010); 36,962,415 (2011); 36,484,846 (2012); 35,597,792 (2013); 35,358,818 (2014); 35,769,942 (2015); 35,675,421 (2016). Statistics based on estimates with a relative SE (SE/weighted estimate) >0.30 or with SE 0 in the nationwide statistics (NIS, Nationwide Emergency Department Sample, and Kids’ Inpatient Database) are not reliable. In 2012, the National Inpatient Sample was redesigned to optimize national estimates. The nationwide statistics in HCUPnet for years before 2012 were regenerated using new trend weights to permit longitudinal analysis. The regenerated data were posted to HCUPnet on July 2, 2014. The statistics for years before 2012 currently on HCUPnet will differ slightly from statistics obtained before July 2, 2014. Information about the NIS redesign and trend weights is available at https://hcupnet.ahrq.gov. For more information about HCUP data, see http://www.hcup-us.ahrq.gov. ICD-9- International Classification of Diseases, Ninth Revision; ICD-10, International Classification of Diseases, Tenth Revision; HCUP, Healthcare Cost and Utilization Project; NIS, National Inpatient Sample. †Because of the transition from ICD-9-CM to ICD-10-CM in October 2015, the data represent 2 time periods. ICD-9 data reflect 2010 through the third quarter of 2015 (2015q3), and ICD-10 data represent the fourth quarter of 2015 (2015q4) through 2016. ‡Cost data were calculated for primary diagnosis only. ICD-9 charge data were obtained solely from HCUP (http://www.hcup-us.ahrq.gov). The aggregate national bill was determined by calculating the mean total charges per year multiplied by number of cases. §Statistics that are based on estimates with a relative SE (SE/weighted estimate) >0.30 or a total cell count <10 in the NIS are not reliable. These statistics are suppressed per HCUP policies.

Babesiosis-related deaths were significantly associated with acute renal failure (p<0.001), acute respiratory failure (p<0.001), and disseminated intravascular coagulation (p = 0.001) when compared with nonfatal hospitalizations. The mean length of stay was 5.8 + 7.3 days (Table 3). The aggregate national bill for the 7-year period for a principal diagnosis of babesiosis was >$170 million USD ($171,281,170), averaging $24.4 million USD per year, and the mean hospital charge for a *Babesia*-associated admission was $36,850.

At least 1 erythrocyte transfusion was reported in 1,560 (20%) hospital admissions (Table 3). Transfusion of other blood products was comparatively rare. Hospitalizations in which erythrocyte transfusions were reported were associated with severe illness. Major or extreme severity of illness was reported in ≈80% in the erythrocyte transfusion group, compared with 53% in those for which erythrocyte transfusions were not reported. Furthermore, 59.9% of the cases in the erythrocyte transfusion group were assessed as having a major or extreme risk for death in comparison with 32.3% for those in which no erythrocyte transfusion was reported. A significantly higher rate of death was observed in the transfusion group (3.18% vs. 1.27%; p = 0.02). Erythrocyte exchange (i.e., erythrocytapheresis) was performed in 80 (1%) admissions. Most admissions in which erythrocytapheresis was undertaken were associated with high illness severity. Specifically, 18.3% were associated with moderate, 18.8% with major, and 63% with extreme severity of illness.

A total of 1,953 (25%) babesiosis-related hospitalizations listed Lyme disease as a concurrent diagnosis; 276 (3.5%) listed Lyme disease as a primary diagnosis. Neither disease severity nor mortality differed for those hospitalizations in which only babesiosis was listed, compared with those hospitalizations in which both babesiosis and Lyme disease were listed (p>0.05). Rates of respiratory failure, heart failure, disseminated intravascular coagulation, and mean length of stay did not differ between those with and without a concurrent diagnosis of Lyme disease.

Other notable concurrent diagnoses in babesiosis-associated hospitalizations were anaplasmosis and ehrlichiosis (658 [8.4%]); these 2 entities are combined because it was not possible to distinguish them on the basis of the coding in use. Malaria, a clinical and morphologic diagnostic mimic of babesiosis, was reported in 52 (0.7%) babesiosis-associated hospitalizations. In 560 (7.2%) of babesiosis-associated hospitalizations, the patients were noted to have decreased splenic function or were asplenic.

When we restricted the hospitalizations to those in which babesiosis was listed in the top 5 diagnoses and a primary diagnosis of Lyme disease was excluded, the number of admissions changed from 7,818 to 6,903. However, all analyses remained comparable (Appendix Tables 1–3).

## Discussion

Our findings offer a nationally representative estimate of in-hospital babesiosis in the United States. During a 7-year period, most (≈85%) *Babesia*-related hospitalizations occurred in the New England and Mid-Atlantic states, and two thirds occurred in the summer (June–August). More than half of all patients were >65 years of age, and almost two thirds were male. A concurrent diagnosis of Lyme disease was reported in one quarter of all babesiosis-related hospitalizations. Reported clinical complications, notably acute renal failure, were common. Consistent with a selection for severe cases that warrant hospital admission, a high proportion of patients experienced major or extreme severity of illness and were deemed to be at high risk for death. Nonetheless, the overall mortality rate was low. Despite an upward trend in annual cases of babesiosis that have been reported to CDC, *Babesia*-related hospitalizations appeared stable or modestly increasing during the study period.

The data on geographic distribution, demographics, and seasonality of *Babesia*-related hospitalizations in this report are consistent with what is known about the parasite’s general epidemiology in the United States. Specifically, *B. microti* is widely endemic in the Northeast and upper Midwest. *B. microti* is the most common species causing human babesiosis in the United States and worldwide. Other species (e.g., *B. duncani*) and variants (e.g., *B.* divergens–like/MO-1) have been reported in the United States but are comparatively rare (*13*–*16*). Male predominance may be attributable to the spectrum of activities that place humans at risk for tick bite. Advanced age is an established risk factor for severe babesiosis because of possible underlying conditions (e.g., cardiorespiratory disease, immunocompromised), immunosenescence, or age-related differential effect of the parasite on the host (*17*–*19*).

*Babesia* infection is seasonal, as illustrated by the more than two thirds of hospitalizations that occurred in summer. Although few hospitalizations occurred in the spring, one fifth of cases occurred from September through December. This finding could be ascribed to a lag in diagnosis; transmission occurs in late spring and summer (i.e., corresponding to the presence of *Ixodes scapularis* nymphs, the primary tick stage that transmits babesiosis), yet hospitalization follows a period of incubation, symptom onset, and progression. Delayed diagnosis and misdiagnosis further account for hospitalizations in late summer and fall. An additional explanation for delayed presentation may be transmission of *Babesia* spp. attributed to bites from adult ticks, which are active into the fall (*20*). Although *I. scapularis* adults are larger than nymphs, and therefore are often removed before transmission, some bites by adult ticks still go unnoticed.

Our findings provided new insights about the overall health burden of babesiosis in the United States, including the number of cases, the severity of illness, and the financial costs incurred by the disease. The overall rate of severe babesiosis requiring hospital admission is increasing, albeit slowly. These findings complement those of Menis et al. who analyzed US Medicare-related claims pertaining to babesiosis during 2006–2017, thus describing a significant increase, from 4 claims/100,000 beneficiaries in 2006 to 9 claims/100,000 beneficiaries in 2017 (*21*). Since babesiosis was designated as a nationally notifiable disease, the number of states where babesiosis is endemic and where reporting of cases is mandatory has increased (*8*,*9*). However, reporting is still incomplete and is not a requirement across all states. Data from CDC indicate an increase in reported cases, which may reflect both a true increase in the number of cases and an increase in awareness and reporting of the disease. In support of this hypothesis, there was a 94% increase in hospitalized patients with babesiosis from the year before national notification to the year after; there were 1,236 *Babesia-*associated discharges from hospitals in 2011, compared with only 636 in 2010. Given that data from NIS hospitalizations represent only a subset of all *Babesia* infections (the most severe cases), increased physician awareness and reporting may play a larger role than an increase in the number of cases.

A stable or modest increase in severe babesiosis (i.e., hospitalizations) does not correlate well with the general epidemiology of this infection, whereby an increase in cases of babesiosis has been observed over the past 3 decades (*22*). Several factors may contribute to a general increase, such as increased geographic spread of the disease, allied with increased recognition of the disease. A group of investigators have used tick surveillance to estimate the geographic range and disease burden of babesiosis (*23*–*26*). A close association between *B. microti* in ticks and reported rates of human infection was demonstrated in babesiosis-endemic areas but not in areas of emerging disease, suggesting underreporting outside of established areas of endemicity (*25*). Furthermore, that the overall reported cases (which included the hospitalized patients) were only ≈50% more may suggest that cases are either not reported or not recognized.

We found that clinical complications of babesiosis in our study population were common, but the overall mortality rate (≈1.3%) was low. The observed rates of clinical complications in our study differ from prior reports, which tended to cite pulmonary sequelae (e.g., pulmonary edema, acute respiratory failure, and acute respiratory distress syndrome) as the most common severe complication of babesiosis (prevalence ranging from 6.3% to 43%) (*17*,*27*–*29*). By contrast, acute renal failure was the leading complication in our population (prevalence of 20.4% for acute renal failure, compared with 6.8% for acute respiratory failure). Previously published prevalence estimates for renal failure have ranged from 4.3% to 7%, the latter in immunocompromised patients (*17*,*27*,*28*). These discrepancies could be due to differences in the definition of organ failure and the scope of prior studies. Many of the reports of organ morbidity are based on small numbers of study participants.

Similarly, previously reported death rates for babesiosis in the United States have been highly variable, from 0% to 27% (*29*,*30*). Differences by reporting source could account for the observed variability. Specifically, most studies that have reported death rates are case series, most of which have been constrained by small sample sizes. There are also differences in the populations that have been described; higher rates have been observed in asplenic patients, immunocompromised persons, and transfusion recipients (*2*,*28*,*30*). Although our study offers a population-based estimate of babesiosis-related death in hospitalized patients, its findings need to be interpreted within the bounds of the acknowledged limitations pertaining to diagnostic coding and clinical imputability. Thus, data are insufficient to conclude whether death rates for babesiosis are improving; rather, the low death rate found in our study may provide a false sense of optimism regarding the disease.

Not surprisingly, erythrocyte transfusion, which was reported in one fifth of hospitalizations, was associated with a significantly higher death rate (≈3%). Erythrocyte transfusions are administered for severe anemia, so they are an index of severe disease, especially for transfusion-transmitted babesiosis, which carries a high death rate (19%) (*2*). Another potential risk factor for severe babesiosis is concurrent Lyme disease; previous studies have found that patients with both babesiosis and Lyme disease have more symptoms that last longer than do those with Lyme disease alone (*31*–*33*). However, the same studies failed to find a difference in the number of symptoms in patients with babesiosis and Lyme disease (i.e., co-infection), compared with patients with babesiosis alone; our data were consistent with those findings. As noted in this analysis, death was a more common outcome in admissions in which babesiosis was listed as a primary rather than as a secondary diagnosis. At least some of the secondary diagnoses are likely to be cases in which the infection was detected incidentally, where parasitemia would likely be low.

The medical care costs of babesiosis also add to the health burden of the disease. The observed charges are comparable to those associated with hospitalizations for Lyme disease; that is, the average hospital charge for a primary diagnosis of Lyme disease was $25,025.53–$31,209.36 during the study period, depending on the reporting period (ICD-9 vs. ICD-10) (data not shown). Although Lyme disease poses a greater health and economic burden, given a greater number of cases and persistence of complications of illness, deaths from Lyme disease, unlike babesiosis, are rarely encountered (*34*).

A limitation of our study is that the analysis is confined to hospitalized case-patients. By design, hospitalizations offer insight into the most severe cases (i.e., there is an inherent selection bias favoring severe infection). Although our findings are informative, quantifying the complete burden of disease given this highly selected sampling is difficult. Furthermore, the sampling approach does not include all hospitalized patients, nor does it include all hospitals; however, it is a validated, robust sampling approach, correlating well with other national survey methods (*35*,*36*). The NIS database is not designed to capture all pediatric patients, although children who are treated in adult hospitals may be captured in these analyses. There are also technical limitations. We cannot determine whether each hospitalization was for a unique patient; a proportion may be readmissions for the same patients, although readmissions are not expected to be common. The analysis also leaves some uncertainty surrounding the relationship between babesiosis and possible concurrent diseases. For example, the observed prevalence of anaplasmosis and ehrlichiosis may reflect infection with *Ehrlichia muris* (also vectored by *I. scapularis* ticks), exposure to similar tick habitats where *I. scapularis* and *Amblyomma* ticks co-exist, or a misdiagnosis. Another limitation is the estimation of hospitalizations for transfusion-transmitted babesiosis. Unfortunately, rates of transfusion-transmitted babesiosis cannot be quantified because causality cannot be established; although one can deduce whether a transfusion was administered, it is not possible to determine whether infection was ascribed to the index transfusion using the database alone. Given the nature of this analysis, we have been cautious not to overinterpret the findings. For example, we cannot be certain whether admissions are increasing in general, whether the demographics of those who are likely to require hospitalization for babesiosis is increasing (i.e., “baby boomers”), or whether babesiosis is simply being recognized in patients because of greater awareness of symptoms.

In conclusion, we found that there has been a modest increase in hospitalized patients with babesiosis in the United States, yet the associated death rate appears to be low. Nonetheless, the overall health burden, particularly for selected patient subsets who are at risk for severe or even fatal disease, remains a concern.

Appendix Additional information on epidemiology of hospitalized patients with babesiosis, United States, 2010–2016.
