# Supplementary material for: Epidemiology of Hospitalized Patients with Babesiosis, United States, 2010–2016
Source: Emerg Infect Dis. 2022 Feb;28(2):354–62. doi: 10.3201/eid2802.210213 (PMC8798708; doi:10.3201/eid2802.210213)
Supplement: Appendix — Additional information on epidemiology of hospitalized patients with babesiosis, United States, 2010–2016. [file 21-0213-Techapp-s1.pdf]

Article DOI: https://doi.org/10.3201/eid2802.210213

Epidemiology of Hospitalized Patients with Babesiosis, United States, 2010–2016

Appendix

Appendix Table 1. Sensitivity analysis comparing the characteristics of hospitalizations in which babesiosis was listed as 1 of all diagnoses vs. hospitalizations in which babesiosis was listed in the top 5 diagnoses and a primary diagnosis of Lyme disease was excluded, National Inpatient Sample, 2010–2016*

Characteristic	ICD-9 and ICD-10 data	ICD-9 and ICD-10 data with babesiosis restricted to top 5 diagnoses and primary Lyme disease excluded
Total, no. (%)	7818 (100)	6903
Demographics		
Age, y, no. (%)		
0-17	30 (0.4)	25 (0.4)
18-44	831(10.6)	681 (9.9)
45-64	2583(33.0)	2321 (33.6)
≥65	4374(55.9)	3876 (56.1)
Mean age	64.7 SD =16.0	65.1 SD=15.8
Median age (IQR)	67 (55-77)	67 (55-77)
Sex, no. (%)		
M	5001 (64.0)	4487 (65.0)
F	2817 (36.0)	2416 (35.0)
Race/ethnicity, no. (%)		
White	6024 (80.1)	5292 (79.7)
African American	245 (3.3)	210 (3.2)
Hispanic	503 (6.7)	454 (6.8)
Asian/Pacific Islander	240 (3.2)	215 (3.2)
Other	509 (6.7)	465 (7.0)
Hospital characteristic		
Elective vs. nonelective admissions, no. (%)		
Nonelective	7452 (95.4)	6581 (95.5)
Elective	356 (4.6)	312 (4.5)
Region of hospital		
Northeast	6140 (86.0)	5443 (86.5)
Midwest	476 (6.7)	426 (6.8)
South	375 (5.3)	316 (5.0)
West	150 (2.1)	110 (1.7)
Hospital bed size		
Small	2159 (30.2)	1981 (31.5)
Medium	2084 (29.2)	1813 (28.8)
Large	2898 (40.6)	2502 (39.7)
Hospital teaching status		
Rural	612 (8.6)	535 (8.5)
Urban nonteaching	2488 (34.8)	2214 (35.2)
Urban teaching	4041 (56.6)	3547 (56.3)

*ICD-9, International Classification of Diseases, Ninth Revision; ICD-10, International Classification of Diseases, Tenth Revision; IQR, interquartile range.

Appendix Table 2. Sensitivity analysis comparing disease severity and comorbid conditions in hospitalizations in which babesiosis was listed as 1 of all diagnoses vs. hospitalizations in which babesiosis was listed in the top 5 diagnoses and a primary diagnosis of Lyme disease was excluded, National Inpatient Sample, 2010–2016*

Characteristic	ICD-9 and ICD-10 data, No. (%)	ICD-9 and ICD-10 data with babesiosis restricted to top 5 diagnoses and primary Lyme disease excluded, no. (%)
APDRG severity of illness		
Minor	376 (4.8)	332 (4.8)
Moderate	2863 (36.6)	2720 (39.4)
Major	3660 (46.8)	3302 (47.8)
Extreme	914 (11.7)	550 (8.0)
APDRG risk for mortality		
Minor	2004 (25.6)	1876 (27.2)
Moderate	2852 (36.5)	2669 (38.7)
Major	2178 (27.9)	1913 (27.7)
Extreme	779 (10.0)	446 (6.5)
Other comorbidities		
Decreased splenic function or Asplenia	560 (7.2)	511 (7.4)
HIV-positive	20 (0.3)	16 (0.2)
Sickle cell disease	30 (0.4)	20 (0.3)
Lyme disease	1953 (25.0)	1458 (21.1)
Primary diagnosis Lyme disease	276 (3.5)	0
Ehrlichiosis and anaplasmosis	658 (8.4)	541 (7.8)
Malaria	52 (0.7)	31 (0.4)
Rocky mountain spotted Fever/rickettsial illness	25 (0.1)	20 (0.3)

*APDRG, All Patient Refined Diagnosis Related Group; ICD-9, International Classification of Diseases, Ninth Revision; ICD-10, International Classification of Diseases, Tenth Revision.

Appendix Table 3. Sensitivity analysis comparing clinical outcomes and healthcare use in hospitalizations in which babesiosis was listed as 1 of all diagnoses vs. hospitalizations in which babesiosis was listed in the top 5 diagnoses and a primary diagnosis of Lyme disease was excluded, National Inpatient Sample, 2010–2016.

Characteristic	ICD-9 and ICD-10 data,* 2010–2016, No. (%)	ICD-9 and ICD-10 data with babesiosis restricted to top 5 diagnoses and primary Lyme disease excluded
Mortality, no. (%)	128 (1.6)	64 (0.9)
Length of stay, d		
Mean (SD)	5.8 (7.3)	5.3 (6.0)
Median (IQR)	4 (3–7)	4 (3–6)
Transfusion and pheresis use, no. (%)		
Erythrocyte transfusion	1560 (20.0)	1336 (19.4)
Platelet transfusion	208 (2.7)	158 (2.3)
Plasma transfused	88 (1.1)	49 (0.7)
Erythrocyte exchange	80 (1.0)	60 (0.9)
Exchange (coded for erythrocytes + plasma)	90 (1.2)	70 (1.0)
Medical complications no. (%)		
Acute renal failure	1594 (20.4)	1260 (18.2)
Respiratory failure	528 (6.8)	313 (4.5)
Acute heart failure	270 (3.5)	175 (2.5)
Disseminated intravascular coagulation	149 (1.9)	90 (1.3)

*ICD-9, International Classification of Diseases, Ninth Revision; ICD-10, International Classification of Diseases, Tenth Revision.
